# Circ_0000064 promotes high glucose-induced renal tubular epithelial cells injury to facilitate diabetic nephropathy progression through miR-532-3p/ROCK1 axis

**DOI:** 10.1186/s12902-022-00968-x

**Published:** 2022-03-15

**Authors:** Huanlan Wang, Shenghua Huang, Taotao Hu, Shizhi Fei, Huanqiao Zhang

**Affiliations:** 1 Department of Nephrology, Wuhan No.1 Hospital, Wuhan, 430022 Hubei China; 2 Department of Nephrology, Baoji Central Hospital, No.8 Jiangtan Road, Weibin District, Baoji, 721008 Shaanxi China

**Keywords:** Diabetic nephropathy, High glucose, Circ_0000064, miR-532-3p, ROCK1

## Abstract

**Background:**

Circular RNA (circRNA) has been shown to mediate diabetic nephropathy (DN) development by regulating renal tubular epithelial cells (RTECs) injury. However, the role and mechanism of circ_0000064 in high glucose (HG)-induced RTECs injury have not been fully elucidated.

**Methods:**

Human RTECs (HK-2) were exposed to HG to induce cell injury. Cell oxidative stress was assessed by detecting the levels of oxidative stress-markers. Moreover, cell proliferation and apoptosis were determined by CCK8 assay, EDU assay and flow cytometry. The protein levels of proliferation markers, apoptosis markers and Rho-associated coiled-coil-containing kinase 1 (ROCK1) were measured using western blot analysis. Furthermore, quantitative real-time PCR was performed to assess the expression of circ_0000064, microRNA (miR)-532-3p and ROCK1. The interaction between miR-532-3p and circ_0000064 or ROCK1 was confirmed by dual-luciferase reporter assay and RNA pull-down assay.

**Results:**

Our results revealed that HG treatment could promote HK-2 cells oxidative stress, apoptosis, fibrosis, and inhibit proliferation. Circ_0000064 expression was increased in the serum of DN patients and HG-induced HK-2 cells, and silenced circ_0000064 could relieve HG-induced HK-2 cells injury. MiR-532-3p could be sponged by circ_0000064, and its overexpression also alleviated HG-induced HK-2 cells injury. Besides, the regulation of circ_0000064 knockdown on HG-induced HK-2 cells injury could be reversed by miR-532-3p inhibitor. Additionally, ROCK1 was a target of miR-532-3p, and its expression was inhibited by circ_0000064 knockdown. The inhibition effect of circ_0000064 knockdown on HG-induced HK-2 cells injury also could be reversed by overexpressing ROCK1.

**Conclusion:**

In summary, circ_0000064 knockdown might alleviate HG-induced HK-2 cells injury via regulating the miR-532-3p/ROCK1 axis, which provided a new perspective for DN treatment.

**Supplementary Information:**

The online version contains supplementary material available at 10.1186/s12902-022-00968-x.

## Highlights

1. Circ_0000064 knockdown relieves HG-induced HK-2 cells injury.
2. Circ_0000064 acts as a sponge of miR-532-3p.
3. MiR-532-3p directly targets ROCK1.

**Supplementary Information:**

The online version contains supplementary material available at 10.1186/s12902-022-00968-x.

## Background

Diabetic nephropathy (DN) is the most common microvascular complication in diabetic patients and is related to metabolic abnormalities caused by hyperglycemia [1, 2]. Recently, the incidence of DN has been increasing year by year, and it has become one of the important causes of death in diabetic patients [3, 4]. It is of great social significance to clarify DN pathogenesis and delay its development. Abnormal function of renal tubular epithelial cells (RTECs) are the key joint leading to DN, and its apoptosis can lead to abnormal tubular reabsorption and renal fibrosis, thereby accelerating the progression of DN [5, 6]. Therefore, elucidating the molecular mechanism that affects the RTECs injury is essential for seeking potential therapeutic targets for DN.

Circular RNA (circRNA) is a non-coding RNA formed by the covalent closure of the 5’end and 3’end of the precursor mRNA [7, 8]. Highly abundant and evolutionarily conserved circRNA has been found to play a vital role in cardiovascular diseases, neurological diseases and cancer [9, 10]. Current research believes that circRNA has powerful biological functions. It can sponge microRNAs (miRNAs) to indirectly regulate mRNA expression, which is called competitive endogenous RNA (ceRNA) function [11, 12]. At present, many circRNAs have been found to regulate the injury of RECTs. For example, circHIPK3 could promote high glucose (HG)-induced HK-2 cells proliferation and inhibit apoptosis to alleviate DN progression [13]. Circ_0003928 was discovered to enhance the apoptosis and inflammatory cytokines secretion of HK-2 cells induced by HG, confirming that circ_0003928 might facilitate DN progression [14].

Circ_0000064 is a newly discovered circRNA that plays an important role in DN development. In the past research, circ_0000064 knockdown was found to inhibit HG-induced mesangial cells oxidative stress, inflammation and extracellular matrix accumulation [15]. Circ_0000064 also had a positively regulation on mesangial cells proliferation and fibrosis [16]. However, it is not clear whether circ_0000064 can also mediate the progression of DN by regulating RECTs injury. Past research pointed out that miR-532-3p was significantly downregulated in many renal progressive diseases, including DN, and its expression was related to the activity of apoptotic pathways [17]. Rho-associated coiled-coil-containing kinase 1 (ROCK1) is a protein serine/threonine kinase that has been shown to be involved in a variety of biological functions in cells [18]. Many studies have shown that ROCK1 is significantly expressed in DN patients and is involved in the regulation of DN progression [19–21].

Here, our study clarified the role and mechanism of circ_0000064 in DN development through HG-induced RECTs injury. According to our research, we found that circ_0000064 could sponge miR-532-3p and miR-532-3p could target ROCK1. Therefore, we proposed and verified the hypothesis that circ_0000064/miR-532-3p/ROCK1 axis regulates DN progression.

## Materials and methods

### Serum samples

37 DN patients and 37 normal healthy volunteers (undergoing physical examination) were recruited from Wuhan No.1 Hospital. DN patients were divided into 2 groups: microalbuminuria group (30 mg/24 h < UAER < 300 mg/24 h) and macroalbuminuria group (UAER > 300 mg/24 h). The clinical characteristics of DN subjects was shown in Table 1. The bloods of patients and normal healthy volunteers were collected and centrifuged to obtain serum samples. For our research, each participant signed the informed consent. Our research was approved by the Ethics Committee of Wuhan No.1 Hospital and was performed in accordance with the Declaration of Helsinki.

**Table 1:** Characteristics of the participants stratified

Clinical parameter	normal	microalbuminuria	macroalbuminuria
Age (years)	51.6 ± 10.5	60.1 ± 11.8	65.9 ± 12.8
Duration of diabetes (years)	5 (1, 8)	9 (4, 14)	12 (9, 22)
BMI (kg/m^2^)	24.1 (22.4, 25.9)	24.8 (22.5, 27.1)	25.1 (23.1, 26.1)
SBP (mmHg)	133 ± 19	147 ± 20	151 ± 22
DBP (mmHg)	82 ± 12	86 ± 13	84 ± 11
PP (mmHg)	49 (42, 58)	55 (44, 66)	66 (48, 78)
FPG (mmol/L)	7.68 (6.32, 9.61)	8.66 (6.32, 10.25)	8.96 (6.65, 10.95)
eGFR (ml/min/1.73m^2^)	101.2 (92.3, 108.5)	89.2 (80.5, 96.8)	52.3 (41.5, 59.6)
UACR (mg/g)	8 (5, 13)	99 (62, 149)	411 (198, 771)
TG (mmol/L)	1.55 (1.01, 2.15)	1.75 (1.98, 2.36)	1.76 (1.89, 2.59)
TC (mmol/L)	4.81 (4.01, 5.63)	5.12 (4.65, 5.96)	5.01 (4.33, 5.75)

*BMI* body mass index, *SBP* Systolic blood pressure, *DBP* Diastolic blood pressure, *PP* Pulse pressure, *FPG* Fasting plasma glucose, *eGFR* Estimated glomerular filtration rate, *UACR* Urinary albumin-to-creatinine ratio, *TG* Triglycerides, *TC* Total cholesterol

### Cell culture and transfection

Human RTECs (HK-2; ATCC, Manassas, VA, USA) were cultured in DMEM medium (Gibco, Grand Island, NY, USA) supplemented with 10% FBS (Gibco) and 1% penicillin/streptomycin (Invitrogen, Carlsbad, CA, USA) at 37 °C with 5% CO_2_. For confirming the effect of HG on the biological functions of HK-2 cells, HK-2 cells were cultured under normal glucose (NG, 5.6 mM D-glucose; Sigma-Aldrich, St. Louis, MO, USA), HG (30 mM D-glucose) and high-mannitol (5.6 mM D-glucose + 24.4 mM mannitol) conditions for 24 h as previously described [22, 23].

### Cell transfection

The small interference RNA of circ_0000064 (si-circ_0000064), miR-532-3p mimic or inhibitor (miR-532-3p or anti-miR-532-3p), pcDNA3.1 ROCK1 overexpression vector, the siRNA of ROCK1 (si-ROCK1), and their negative controls were synthesized from Genepharma (Shanghai, China). Lipofectamine 3000 (Invitrogen) was used to transfect the siRNA (50 nM), miRNA mimic (50 nM), miRNA inhibitor (50 nM) and vectors (4.0 μg) into HK-2 cells. After transfection for 24 h, cells were treated with HG for 24 h.

### Determination of SOD and MDA levels

The levels of SOD and MDA in the lysates of HK-2 cells were measured to assess cell oxidative stress according to the instructions of SOD and MDA Assay Kits (Shanghai Enzyme-linked Biotechnology Co., Ltd., Shanghai, China), respectively.

### Cell counting kit 8 (CCK8) assay

After transfection or treatment, HK-2 cells were harvested and re-seeded into 96-well plates. After cultured for 48 h, 10 μL CCK8 solution (Dojindo, Kumamoto, Japan) was added into cells and incubated for 4 h. Finally, the optical density (OD) was measured by a microplate reader at 450 nm to assess cell viability.

### EDU assay

EDU Cell Proliferation Kit (Beyotime, Shanghai, China) was used to measure cell proliferation. In brief, HK-2 cells in 6-well plates were cultured for 48 h. Then, cells were treated with EDU solution for 2 h, Click Reaction Buffer for 30 min and DAPI solution for 10 min. Under a fluorescence microscope, cell fluorescence images were obtained and EDU positive cells were counted using Image J software.

### Western blot (WB) analysis

RIPA lysis buffer (Beyotime) was used to extract total protein. 30 μg protein was separated by 10% SDS-PAGE gel and transferred to PVDF membrane followed by hatched with non-fat milk. Afterwards, the membranes were treated with anti-PCNA (1:1000, ab18197, Abcam, Cambridge, MA, USA), anti-cyclin D1 (1:1000, ab16663, Abcam), anti-Bax (1:2000, ab32503, Abcam), anti-Bcl2 (1:5000, ab182858, Abcam), anti-ROCK1 (1:500, ab134181, Abcam), anti-cleaved-caspase3 (1:1000, ab2302, Abcam), anti-α-SMA (1:1000, ab5694, Abcam), anti-collagen I (1:1000, ab34710, Abcam) or anti-β-actin (1:1000, ab8227, Abcam) followed by incubated with Goat Anti-Rabbit IgG antibody (1:20,000, ab205718, Abcam). BeyoECL Moon Detection Kit (Beyotime) was used to visualize the protein bands and the band density was analyzed by Image J Software.

### Flow cytometry

After transfection or treatment, HK-2 cells were harvested and washed with PBS. After suspended with 1 × binding buffer, cells were then stained with Annexin V-FITC and PI solution in the dark for 15 min in accordance with the instructions of Annexin V-FITC Apoptosis Kit (Biovision, Milpitas, CA, USA). FACScan flow cytometer was used for analyzing, and cell apoptosis rate was analyzed by CellQuest software. For measuring cell cycle process HK-2 cells were fixed with precooled 70% ethanol and stained with RNase A and PI solution for 30 min in line with the instructions of Cell Cycle Detection Kit (Beyotime). Finally, cell cycle distribution was analyzed using FACScan flow cytometry.

### Quantitative real-time PCR (qRT-PCR)

Total RNA from serum samples and HK-2 cells was extracted using RNAiso reagent (TaKaRa, Dalian, China), and cDNA was synthesized by PrimeScript RT Reagent Kit (TaKaRa). QRT-PCR was performed by SYBR Green Reagent (TaKaRa) and specific primers. Fold change was calculated with 2^-ΔΔCT^ method and normalized by β-actin or U6. The primer sequences were shown in Table 2.

**Table 2:** Primer sequences used for PCR

Name		Primers for PCR (5′-3′)
circ_0000064	Forward	CTAGAGGCGGTGGCGTTG
	Reverse	TGAGGTCCCCAGTGCCC
ROCK1	Forward	AAATTGCTTTCCGCTGCTGG
	Reverse	CGCAGCAGGTTGTCCATTTT
miR-532-3p	Forward	GCCGAGCCTCCCACACCCAAGG
	Reverse	CTCAACTGGTGTCGTGGAG
β-actin	Forward	CTTCGCGGGCGACGAT
	Reverse	CCACATAGGAATCCTTCTGACC
U6	Forward	CTCGCTTCGGCAGCACA
	Reverse	AACGCTTCACGAATTTGCGT

### Dual-luciferase reporter assay

According to the binding sites and mutant sites between miR-532-3p and circ_0000064 or ROCK1 3’UTR, the wild-type and mutant-type vectors of circ_0000064 (circ_0000064^WT/MUT^ or ROCK1-3’UTR^WT/MUT^) were constructed using pmirGLO luciferase reporter. HK-2 cells were transfected with miR-532-3p mimic and the above vectors using Lipofectamine 3000. 48 h later, luciferase activity (*Firefly*/*Renilla*) was evaluated using Dual-luciferase Reporter Gene Assay Kit (Beyotime).

### RNA pull-down assay

This assay was used to further confirm the interaction between circ_0000064 and miR-532-3p. In brief, biotin-labeled miR-532-3p probe (Bio-miR-532-3p) and negative control probe (Bio-miR-NC) constructed and synthesized by Genepharma were transfected into HK-2 cells. 48 later, cell lysates were incubated with Dynabeads Streptavidin (Invitrogen), and circ_0000064 enrichment was examined by qRT-PCR.

### Statistical analysis

Data were presented as mean ± SD from 3 independent experiments. Statistical analysis was performed using GraphPad Prism Version 8.0. Differences between groups were analyzed using Student’s *t*-test or ANOVA followed by Tukey’s post-hoc test. *P* < 0.05 was considered significant difference.

## Results

### HG could induce HK-2 cells injury

**Fig. 1:**
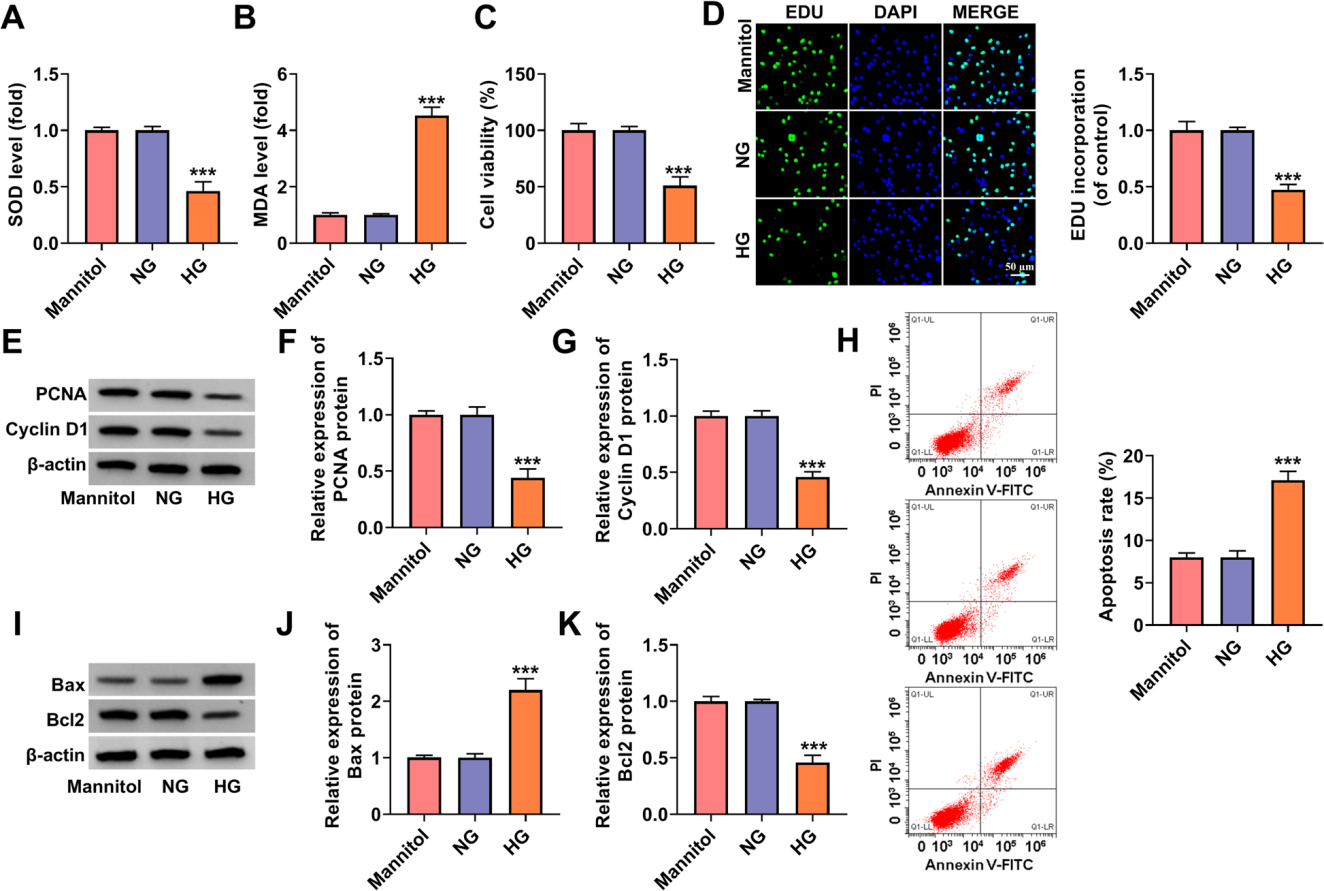
HG could induce HK-2 cells injury. HK-2 cells were cultured under HG, NG or mannitol condition. **A**-**B** Cell oxidative stress was assessed by determining the levels of SOD and MDA. CCK8 assay (**C**) and EDU assay (**D**) were performed to assess cell proliferation. **E**-**G** The protein expression of PCNA and cyclin D1 was measured by WB analysis. **H** Cell apoptosis rate was evaluated by flow cytometry. **I**-**K** WB analysis was used to examine the protein expression of Bax and Bcl2. ***P < 0.001

After HG treatment, we assessed the oxidative stress, proliferation and apoptosis of HK-2 cells. Through detecting the levels of oxidative stress markers, we found that SOD level was reduced and MDA level was enhanced in HG-treated HK-2 cells compared to the NG group and mannitol group (Fig. 1A-B). Further analysis showed that HG could inhibit the viability and the EDU positive cells (Fig. 1C-D), as well as decease the protein expression of proliferation markers PCNA and cyclin D1 (Fig. 1E-G). In addition, HG treatment also enhanced cell apoptosis rate, increased apoptosis marker Bax and cleaved-caspase3 protein expression, promoted fibrosis marker α-SMA and collagen I protein expression, and deceased anti-apoptosis marker Bcl2 protein expression in HK-2 cells (Fig. 1H-K and Supplementary Fig. 1A). These data indicated that HG could promote the oxidative stress and apoptosis, while inhibit the proliferation of HK-2 cells, confirming that HG could induce HK-2 cells injury.

### Circ_0000064 was overexpressed in DN patients and HG-induced HK-2 cells

**Fig. 2:**
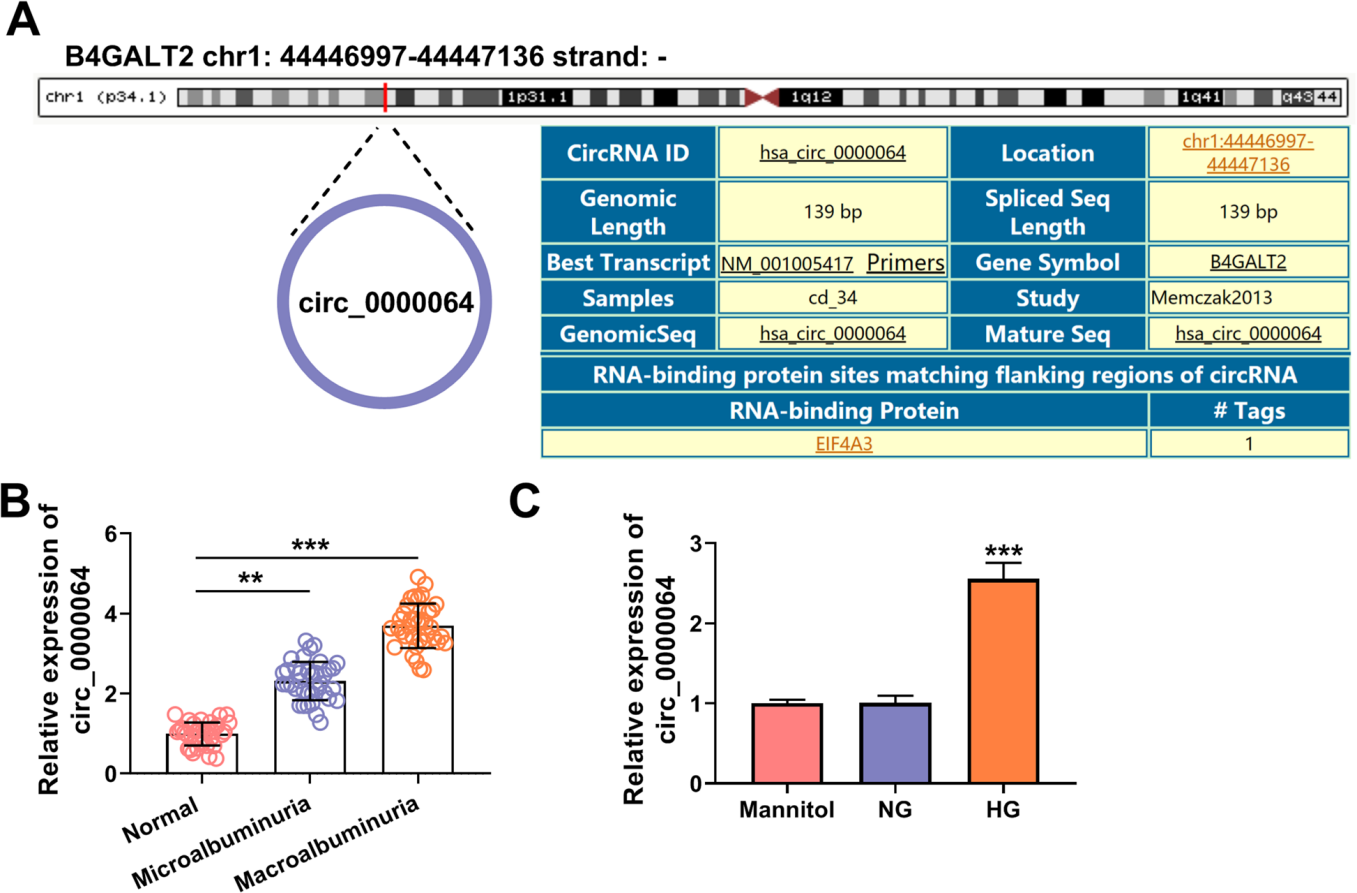
Circ_0000064 was overexpressed in DN patients and HG-induced HK-2 cells. **A** The basic information of circ_0000064 was shown. **B** The expression of circ_0000064 in the serum of DN patients with microalbuminuria or macroalbuminuria and normal healthy volunteers was measured by qRT-PCR. **C** QRT-PCR was performed to detect circ_0000064 expression in HK-2 cells under HG, NG or mannitol condition. ***P < 0.001

Circ_0000064, located at chr1: 44446997-44,447,136, has a length of 139 bp and is derived from B4GALT2 genic (Fig. 2A). By detecting the expression of circ_0000064 in the serum of DN patients and normal healthy volunteers, we confirmed that circ_0000064 was upregulated in DN patients with microalbuminuria and was higher in macroalbuminuria patients (Fig. 2B). Besides, the highly expressed circ_0000064 also was found in HG-induced HK-2 cells compared to the NG group and mannitol group (Fig. 2C). This suggested that the high expression of circ_0000064 might be related to the progression of DN.

### Circ_0000064 knockdown alleviated HG-induced HK-2 cells injury

**Fig. 3:**
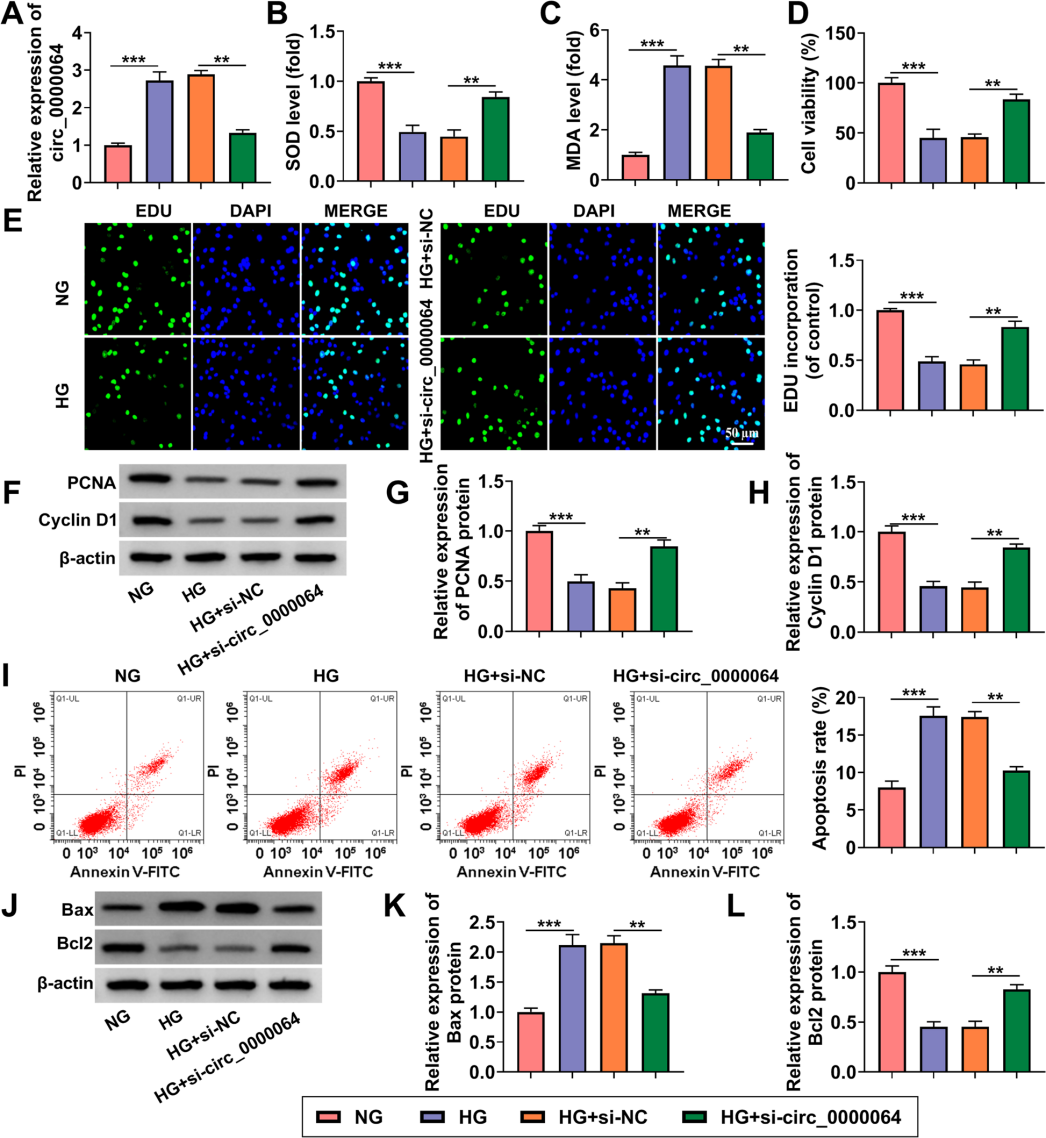
Circ_0000064 knockdown alleviated HG-induced HK-2 cells injury. HK-2 cells were transfected with si-NC or si-circ_0000064 and then treated with HG. **A** QRT-PCR was used to measure circ_0000064 expression. **B**-**C** The levels of SOD and MDA were measured to assess cell oxidative stress. CCK8 assay (**D**) and EDU assay (**E**) were used to detect cell proliferation. **F**-**H** WB analysis was utilized to determine the protein expression of PCNA and cyclin D1. **I** Flow cytometry was used to assess cell apoptosis rate. **J**-**L** The protein expression of Bax and Bcl2 was detected by WB analysis. **P < 0.01, ***P < 0.001

To determine the role of circ_0000064 in DN progression, circ_0000064 was silenced in HK-2 cells using si-circ_0000064. The detection of circ_0000064 expression confirmed that si-circ_0000064 indeed reduced circ_0000064 expression in HG-induced HK-2 cells (Fig. 3A). After knockdown of circ_0000064, HG-induced oxidative stress in HK-2 cells could be significantly reduced, showing that SOD level was increased and MDA level was decreased in the HG + si-circ_0000064 group (Fig. 3B-C). Moreover, circ_0000064 silencing could enhance the viability, EDU positive cells and the protein expression of PCNA and cyclin D1 in HG-induced HK-2 cells (Fig. 3D-H). Also, the detection of cell cycle distribution showed that si-circ_0000064 promoted the cell cycle in HG-treated HK-2 cells, while anti-miR-532-3p reversed this effect (Supplementary Fig. 2). Also, downregulation of circ_0000064 inhibited the apoptosis rate and suppressed Bax, cleaved-caspase3, α-SMA and collagen I protein expression, while promoted Bcl2 protein expression in HG-induced HK-2 cells (Fig. 3I-L and Supplementary Fig. 1B). Above all, these results showed that circ_0000064 knockdown could relieve HG-induced HK-2 cells injury, confirming that circ_0000064 might promote DN progression.

### MiR-532-3p could be sponged by circ_0000064

**Fig. 4:**
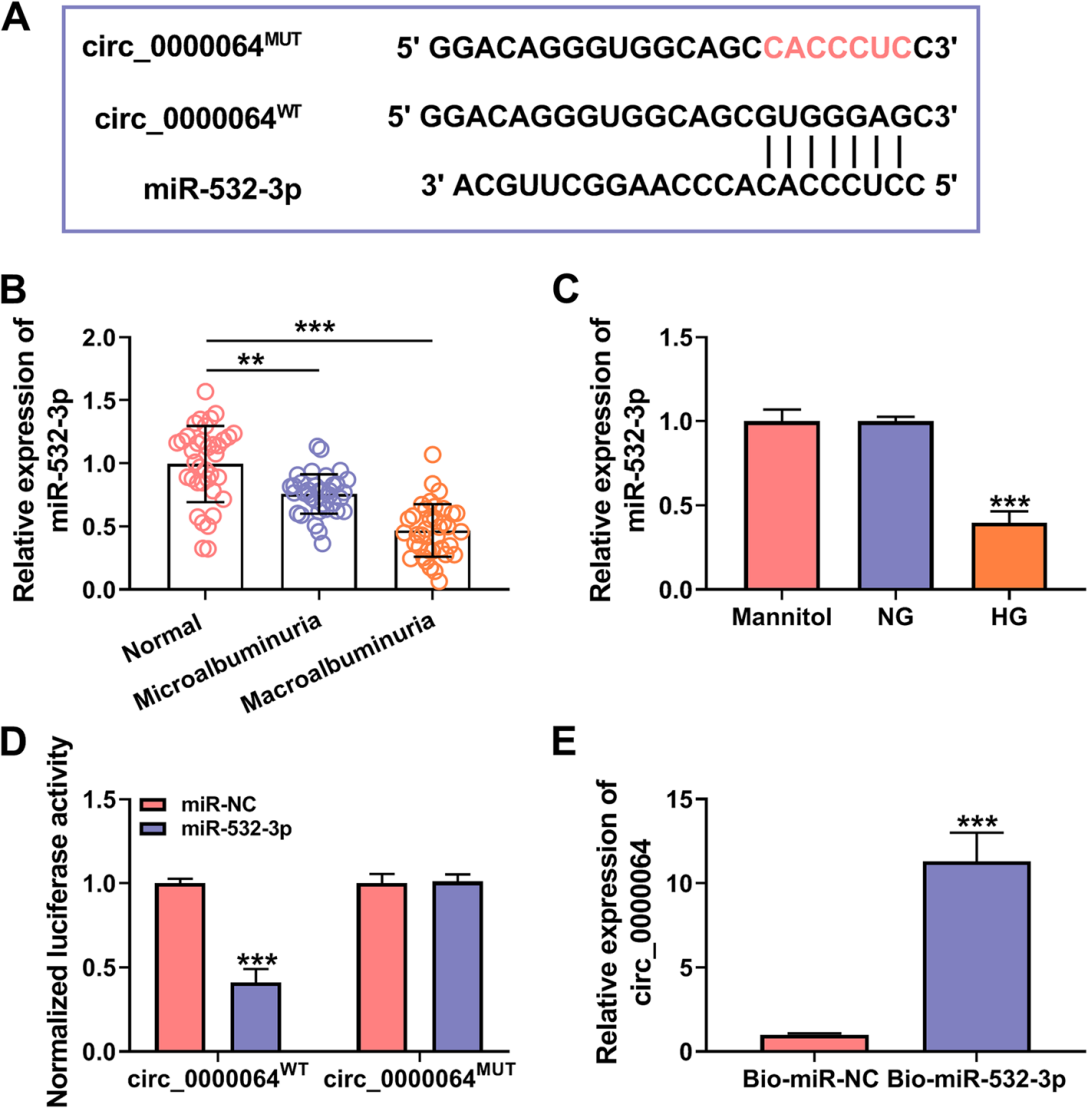
MiR-532-3p could be sponged by circ_0000064. **A** The binding sites and mutant sites between circ_0000064 and miR-532-3p were shown. **B** The expression of miR-532-3p was detected by qRT-PCR in the serum of DN patients with microalbuminuria or macroalbuminuria and normal healthy volunteers. **C** QRT-PCR was used to measure the miR-532-3p expression in HK-2 cells under HG, NG or mannitol condition. Dual-luciferase reporter assay (**D**) and RNA pull-down assay (**E**) were used to assess the interaction between circ_0000064 and miR-532-3p. ***P < 0.001

To explore the mechanism of circ_0000064 regulated DN progression, the circinteractome online software was used to search for the targeted miRNA. As a result, we found the complementary binding sites between miR-532-3p and circ_0000064 (Fig. 4A). MiR-532-3p was discovered to be lowly expressed in the serum of DN patients and HG-induced HK-2 cells (Fig. 4B-C). In addition, dual-luciferase reporter assay and RNA pull-down assay were used to further verify the interaction between miR-532-3p and circ_0000064. The results showed that miR-532-3p mimic could only specifically inhibit the luciferase activity of circ_0000064^WT^ vector (Fig. 4D), and circ_0000064 could be markedly enriched in the Bio-miR-532-3p probe (Fig. 4E). These results revealed that circ_0000064 could sponge miR-532-3p.

### MiR-532-3p relieved HG-induced HK-2 cells injury

**Fig. 5:**
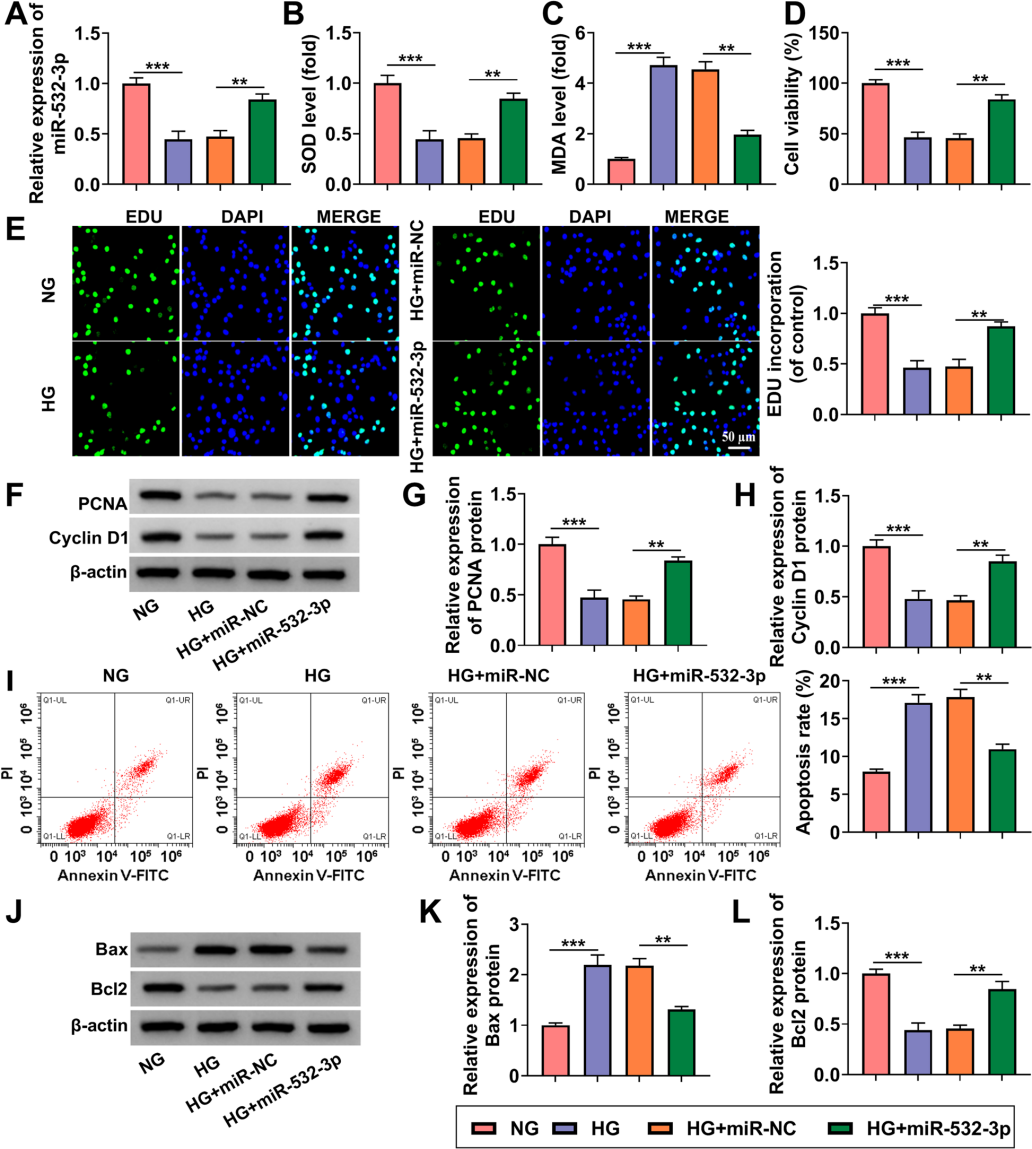
MiR-532-3p relieved HG-induced HK-2 cells injury. HK-2 cells were transfected with miR-NC or miR-532-3p mimic and then treated with HG. **A** The expression of miR-532-3p was detected by qRT-PCR. **B**-**C** Cell oxidative stress was analyzed by detecting the levels of SOD and MDA. Cell proliferation was assessed using CCK8 assay (**D**) and EDU assay (**E**). **F**-**H** The protein expression of PCNA and cyclin D1 was examined using WB analysis. **I** Cell apoptosis rate was analyzed using flow cytometry. **J**-**L** The protein expression of Bax and Bcl2 was determined using WB analysis. **P < 0.01, ***P < 0.001

To confirm the role of miR-532-3p in DN progression, HK-2 cells were transfected with miR-532-3p mimic and then treated with HG. By detecting miR-532-3p expression, we confirmed that miR-532-3p expression was significantly enhanced in HG-induced HK-2 cells (Fig. 5A). Overexpressed miR-532-3p increased SOD level and inhibited MDA level in HG-induced HK-2 cells (Fig. 5B-C). Meanwhile, the proliferation of HK-2 cells induced by HG was markedly promoted by miR-532-3p overexpression, which showed that the cell viability, the EDU positive cells and the protein expression of PCNA and cyclin D1 were increased in the HG + miR-532-3p group (Fig. 5D-H). In addition, miR-532-3p mimic also suppressed the apoptosis rate and the Bax, cleaved-caspase3, α-SMA and collagen I protein expression, while enhanced the Bcl2 protein expression in HG-induced HK-2 cells (Fig. 5I-L and Supplementary Fig. 1C). All data showed that miR-532-3p might play a negative role in DN progression.

### Circ_0000064 regulated HG-induced HK-2 cells injury by sponging miR-532-3p

**Fig. 6:**
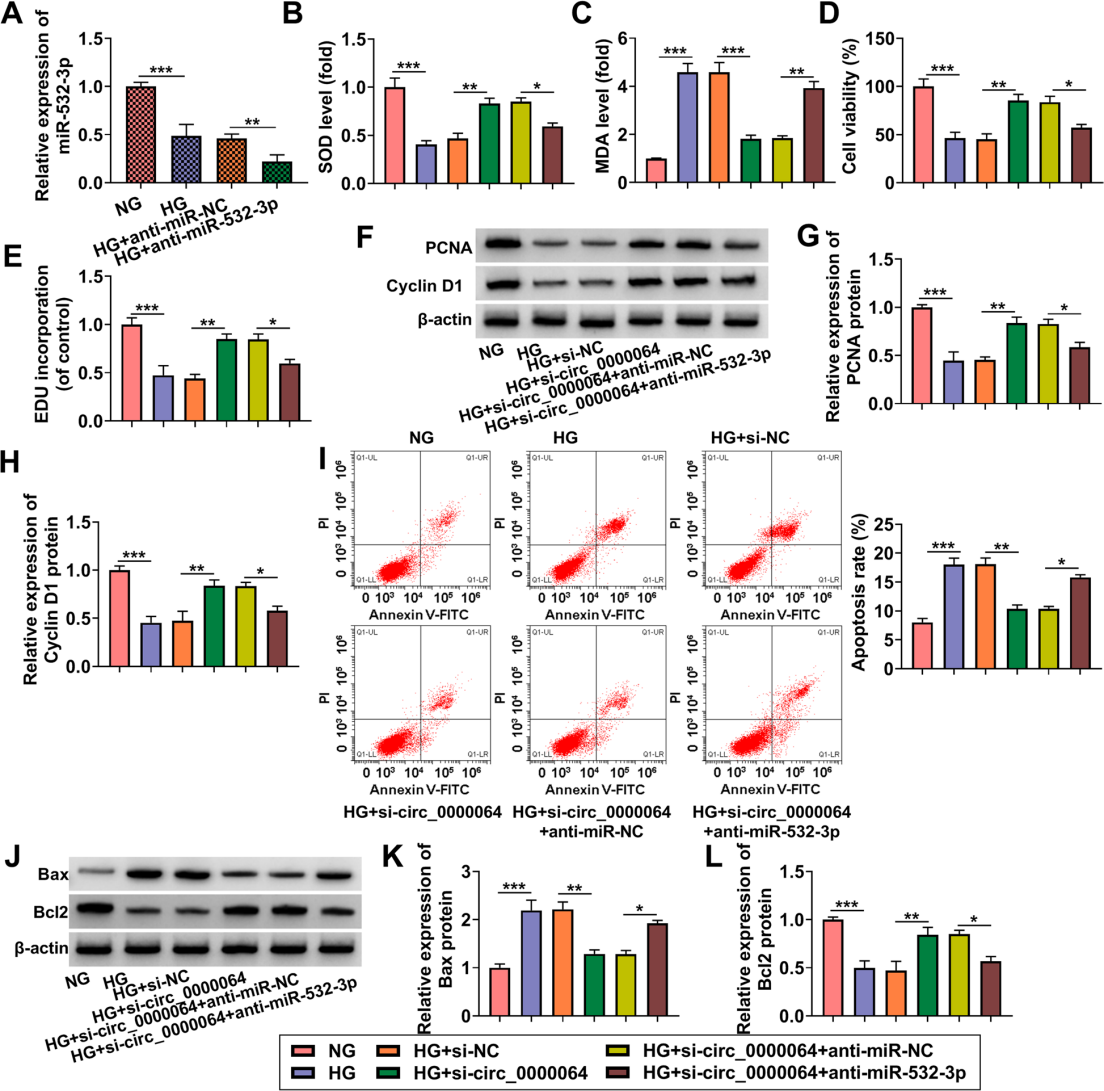
Circ_0000064 regulated HG-induced HK-2 cells injury by sponging miR-532-3p. **A** QRT-PCR analysis was performed to measure miR-532-3p expression in HG-induced HK-2 cells transfected with anti-miR-NC or anti-miR-532-3p. **B**-**L** HK-2 cells were transfected with si-NC, si-circ_0000064, si-circ_0000064 + anti-miR-NC or si-circ_0000064 + anti-miR-532-3p, and then treated with HG. **B**-**C** The levels of SOD and MDA were determined to measure cell oxidative stress. Cell proliferation was analyzed using CCK8 assay (**D**) and EDU assay (**E**). **F**-**H** WB analysis was used to detect the protein expression of PCNA and cyclin D1. **I** Cell apoptosis rate was analyzed by flow cytometry. **J**-**L** The protein expression of Bax and Bcl2 was examined by WB analysis. *P < 0.05, **P < 0.01, ***P < 0.001

To investigate whether circ_0000064 sponged miR-532-3p to regulate DN progression, we performed the rescue experiments. The transfection of anti-miR-532-3p indeed reduced miR-532-3p expression in HG-induced HK-2 cells (Fig. 6A). After that, HK-2 cells were co-transfected with si-circ_0000064 and anti-miR-532-3p followed by treated with HG. Our data showed that the enhancing effect of si-circ_0000064 on SOD level and the suppressive effect on MDA level in HG-induced HK-2 cells could be abolished by miR-532-3p inhibitor (Fig. 6B-C). Meanwhile, miR-532-3p inhibitor also reversed the promotion effects of si-circ_0000064 on cell viability, the EDU positive cells, and the protein expression of PCNA and cyclin D1 in HG-induced HK-2 cells (Fig. 6D-H). Through measuring cell apoptosis rate and the protein expression of Bax, Bcl2, cleaved-caspase3, α-SMA and collagen I, we confirmed that the inhibitory effect of circ_0000064 knockdown on the apoptosis and fibrosis of HG-induced HK-2 cells could be eliminated by miR-532-3p inhibitor (Fig. 6I-L and Supplementary Fig. 1D). Hence, these data showed that circ_0000064 sponged miR-532-3p to regulate DN progression.

### MiR-532-3p targeted ROCK1

**Fig. 7:**
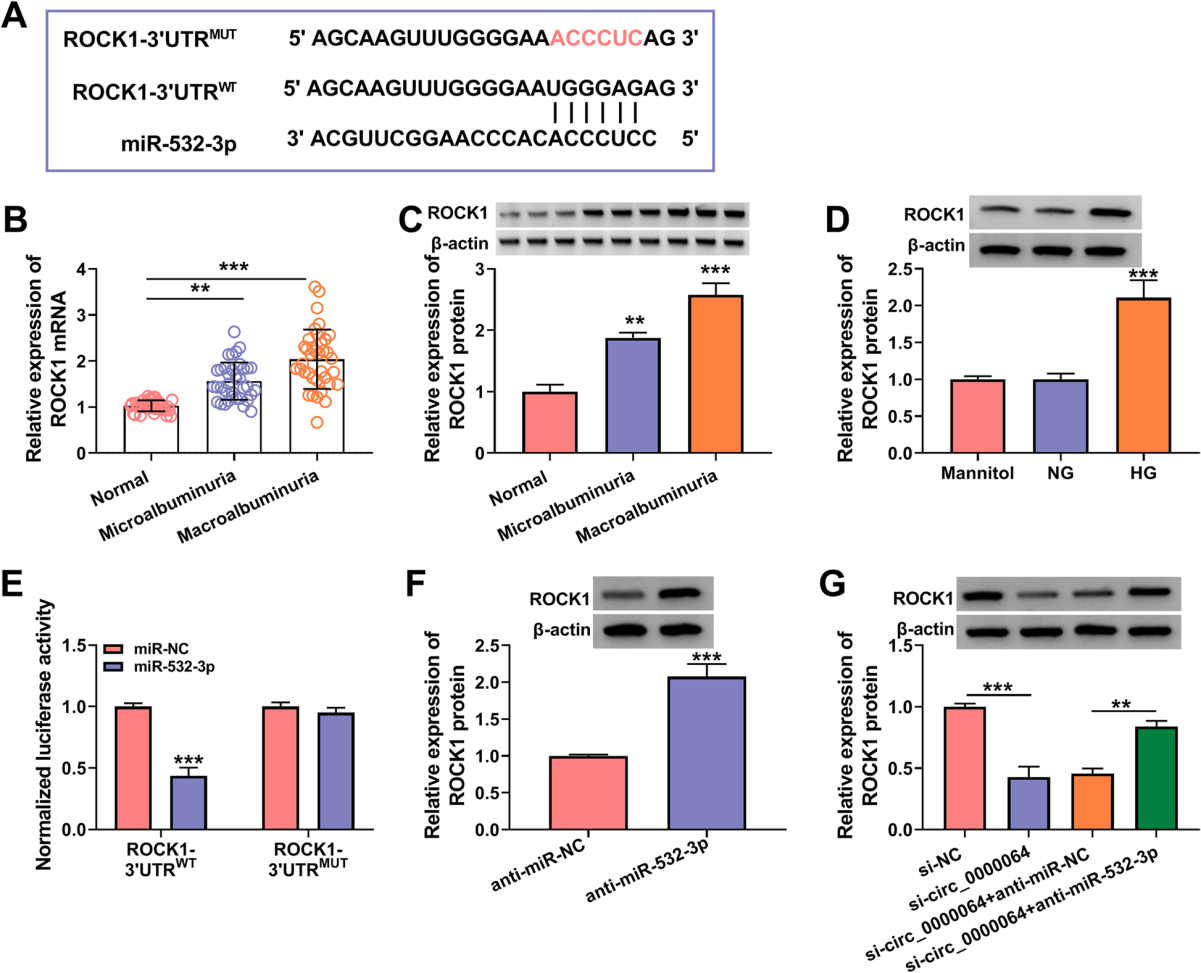
MiR-532-3p targeted ROCK1. **A** The binding sites and mutant sites between miR-532-3p and ROCK1 3’UTR were shown. **B**-**C** The mRNA and protein expression of ROCK1 in the serum of DN patients with microalbuminuria or macroalbuminuria and normal healthy volunteers was examined by qRT-PCR and WB analysis. **D** WB analysis was used to measure the protein expression of ROCK1 in HK-2 cells under HG, NG or mannitol condition. **E** Dual-luciferase reporter assay was used to confirm the interaction between miR-532-3p and ROCK1. **F** ROCK1 protein expression was detected by WB analysis in HK-2 cells transfected with anti-miR-NC or anti-miR-532-3p. **G** WB analysis was used to measure ROCK1 protein expression in HK-2 cells transfected with si-NC, si-circ_0000064, si-circ_0000064 + anti-miR-NC or si-circ_0000064 + anti-miR-532-3p. **P < 0.01, ***P < 0.001

Subsequently, targetscan software was used to predict the target of miR-532-3p, and ROCK1 3’UTR was found to have binding sites with miR-532-3p (Fig. 7A). In the serum of DN patients with microalbuminuria and macroalbuminuria, ROCK1 was significantly upregulated compared to normal healthy volunteers at the mRNA level and protein level (Fig. 7B-C). Besides, ROCK1 protein expression also was markedly increased in HG-induced HK-2 cells (Fig. 7D). Using dual-luciferase reporter assay, we found that miR-532-3p mimic could inhibit the luciferase activity of ROCK1-3’UTR^WT^ vector without affecting that of the ROCK1-3’UTR^MUT^ vector, confirming the interaction between ROCK1 and miR-532-3p (Fig. 7E). After silenced miR-532-3p using anti-miR-532-3p, ROCK1 protein expression was discovered to be upregulated in HK-2 cells (Fig. 7F). Moreover, silenced circ_0000064 remarkably reduced ROCK1 protein expression, while this effect could be reversed by miR-532-3p inhibitor (Fig. 7G). These data illuminated that circ_0000064 could sponge miR-532-3p to positively regulate ROCK1.

### ROCK1 partially reversed the regulation of si-circ_0000064 on HG-induced HK-2 cells injury

**Fig. 8:**
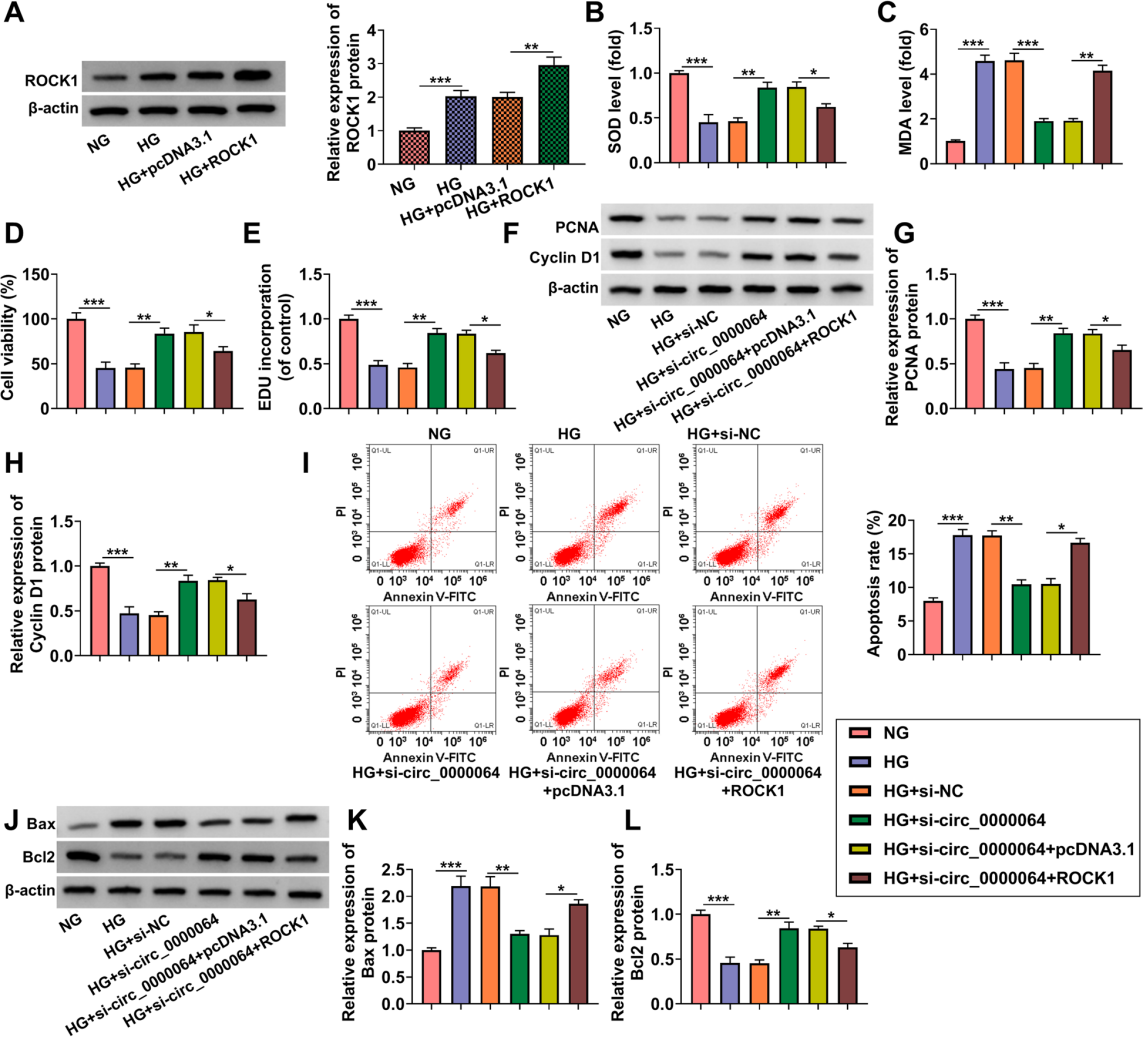
ROCK1 partially reversed the regulation of si-circ_0000064 on HG-induced HK-2 cells injury. **A** WB analysis was used to detect ROCK1 protein expression in HG-induced HK-2 cells transfected with pcDNA3.1 or ROCK1 overexpression vector. **B**-**L** HK-2 cells were transfected with si-NC, si-circ_0000064, si-circ_0000064 + pcDNA3.1 or si-circ_0000064 + ROCK1, and then treated with HG. **B**-**C** Cell oxidative stress was evaluated by measuring the levels of SOD and MDA. Cell proliferation was detected by CCK8 assay (**D**) and EDU assay (**E**). (F-H) The protein expression of PCNA and cyclin D1 was detected using WB analysis. **I** Cell apoptosis rate was determined using flow cytometry. **J**-**L** WB analysis was performed to analyze the protein expression of Bax and Bcl2. *P < 0.05, **P < 0.01, ***P < 0.001

Then, we assessed the effect of ROCK1 in HG-induced HK-2 cells injury. After HK-2 cells were transfected si-ROCK1 and treated with HG, we found that si-ROCK1 inhibited ROCK1 protein expression induced by HG (Supplementary Fig. 3A). The addition of si-ROCK1 also increased SOD level and suppressed MDA level in HG-induced HK-2 cells (Supplementary Fig. 3B-C). ROCK1 knockdown accelerates cell viability, EDU incorporation, and the protein expression of PCNA and Cyclin D1 in HG-induced HK-2 cells (Supplementary Fig. 3D-H). Not only that, downregulation of ROCK1 inhibited cell apoptosis rate and the protein expression of Bax, cleaved-caspase3, α-SMA and collagen I, while increased the Bcl2 protein expression in HG-induced HK-2 cells (Supplementary Fig. 3I-M). These data revealed that ROCK1 might contribute to HG-induced HK-2 cells injury. To further confirm that circ_0000064 regulated DN progression by regulating ROCK1, we performed the rescue experiments. After transfected with ROCK1 overexpression vector into HG-induced HK-2 cells, ROCK1 protein expression was markedly enhanced, confirming the transfection efficiency of ROCK1 overexpression vector (Fig. 8A). Then, HK-2 cells were co-transfected with si-circ_0000064 and ROCK1 overexpression vector followed by treated with HG. Our data showed that overexpressed ROCK1 reversed the increasing effect of si-circ_0000064 on SOD level and the decreasing effect on MDA level in HG-induced HK-2 cells (Fig. 8B-C). Also, the promotion effects of circ_0000064 knockdown on cell viability, the EDU positive cells and the protein expression of PCNA and cyclin D1 in HG-induced HK-2 cells could be abolished by overexpressing ROCK1 (Fig. 8D-H). Additionally, the suppressive effect of si-circ_0000064 on cell apoptosis rate also was reversed by ROCK1 overexpression (Fig. 8I). Furthermore, we also observed that Bax, cleaved-caspase3, α-SMA and collagen I protein expression levels were increased and Bcl2 protein expression was decreased in the co-transfection group (Fig. 8J-L and Supplementary Fig. 1E). Therefore, our data showed that circ_0000064 knockdown alleviated DN progression through inhibiting ROCK1.

## Discussion

The pathogenesis of DN is closely related to hyperglycemia, but its specific mechanism is still unclear. Studies have shown that oxidative injury and apoptosis of RTECs can be observed in the early stage of DN under HG environment [24, 25]. Therefore, RTECs injury is an important cause of DN development. Here, we exposed HK-2 cells to HG conditions to investigate the function of circ_0000064 in HG-induced RTECs injury. Many past studies had shown that circ_0000064 was highly expressed in cancer tissues and promoted the malignant progression of cancers, including gastric cancer [26], lung cancer [27] and hepatocellular carcinoma [28]. Our study showed that circ_0000064 knockdown alleviated HG-induced HK-2 cells oxidative stress, apoptosis and fibrosis, suggesting that circ_0000064 might be a key regulator in promoting DN progression, which was consistent with previous conclusions [15, 16]. This result once again confirmed that targeted inhibition of circ_0000064 might be an effective way to alleviate the progression of DN.

The ceRNA mechanism of circRNA has been confirmed in many studies. At present, circ_0000064 is believed to target miR-30c-5p or miR-143 to regulate the biological function of mesangial cells, thereby mediating the progression of DN [15, 16]. Here, we revealed a novel target miRNA of circ_0000064, miR-532-3p. In past studies, miR-532-3p has been found to play a different role in different types of cancers. Gu et al. showed that miR-532-3p could suppress colorectal cancer proliferation and promote apoptosis to inhibit cancer development [29]. On the contrary, Wang et al. reported that miR-532-3p had a promotion effect on hepatocellular carcinoma proliferation and metastasis [30]. MiR-532-3p had been shown to promote ox-LDL-induced vascular smooth muscle cells proliferation and migration [31]. The dysregulation of miR-532-3p-CSF2RA axis was associated with vulnerable plaque formation [32]. Studies had shown that the expression of circulating miRNAs, including miR-532-3p, could be altered by regular endurance training [33]. A recent study has shown that miR-532-3p could inhibit oxidative stress injury caused by cerebral ischemia/reperfusion, suggesting that it might be a potential target for ischemic stroke treatment [34]. In diabetes, the miR-532-3p expression might be related to chronic inflammation and oxidative stress [35], and it might regulate GLUT/HK2 pathway to participate in insulin resistance [36]. In this, we confirmed that miR-532-3p had a decreased expression in DN patients and HG-induced HK-2 cells. The results of functional experiments suggested that miR-532-3p overexpression promoted HG-induced HK-2 cells proliferation, while inhibited oxidative stress, apoptosis and fibrosis. The anti-oxidative stress injury effect of miR-532-3p was confirmed in our study, which was similar to the previous results [34]. Additionally, the reversal effect of miR-532-3p inhibitor on si-circ_0000064-mediated HK-2 cells injury under HG condition also demonstrated that circ_0000064 sponged miR-532-3p to regulate DN progression.

ROCK1 could promote glomeruli endothelial-mesenchymal transition to enhance albuminuria, thereby aggravating DN progression [19]. Also, knockdown of ROCK1 was confirmed to inhibit mitochondrial fission to alleviate the development of DN [20, 21]. According to the latest research, islet transplantation could relieve podocytes injury via inhibiting ROCK1-related pathway [37]. Here, we confirmed that ROCK1 was targeted by miR-532-3p, and revealed that circ_0000064 sponged miR-532-3p to upregulate ROCK1. After overexpressing ROCK1, we discovered that the si-circ_0000064-mediated HK-2 cells injury could be reversed, which further confirmed that the regulation of circ_0000064 on DN progression was achieved by mediating the expression of ROCK1. Previous studies have shown that the expression of ROCK1 is related to apoptosis and oxidative stress, and the loss of ROCK1 activity can effectively inhibit apoptosis and oxidative stress [38, 39]. In this, we confirmed that ROCK1 had a pro-apoptosis and pro-oxidative stress role in HG-induced HK-2 cells. Above all, our study putted forward the result that the circ_0000064/miR-532-3p/ROCK1 axis mediated the progression of DN.

## Conclusion

In summary, our study showed that circ_0000064 facilitated RTECs injury under HG conditions by targeting the miR-532-3p/ROCK1 axis to promote DN progression. The mitigation effect of si-circ_0000064 on RTECs injury revealed that circ_0000064 might be a potential target for DN treatment. Of course, there are some limitations to our research. Due to limited conditions, we have not carried out in vivo tests to further confirm our conclusions. In future studies, we will build animal models to verify our experimental results.

## Supplementary Information

**Additional file 1: Supplementary Figure 1**. The protein expression of cleaved-caspase3, α-SMA and collagen I. Protein expression was detected under the treatment conditions as shown in Fig. 1 (A), Fig. 3 (B), Fig. 5 (C), Fig. 6 (D) and Fig. 8 (E). **P < 0.01, ***P < 0.001.

**Additional file 2: Supplementary Figure 2**. Effects of si-circ_0000064 and anti-miR-532-3p on cell cycle. HK-2 cells were transfected with si-NC or si-circ_0000064 and then treated with HG. Cell cycle distribution was assessed by flow cytometry. *P < 0.05, **P < 0.01, ***P < 0.001.

**Additional file 3: Supplementary Figure 3**. Knockdown of ROCK1 relieved HG-induced HK-2 cells injury. HK-2 cells were transfected with si-NC or si-ROCK1 and then treated with HG. (A) The ROCK1 protein expression was detected by WB analysis. (B-C) Cell oxidative stress was analyzed by detecting SOD and MDA levels. CCK8 assay (D) and EDU assay (E) were used to assess cell proliferation. (F-H) The protein expression of PCNA and cyclin D1 was tested by WB analysis. (I) Flow cytometry was used to examine cell apoptosis rate. (J-M) The protein expression was determined by WB analysis. **P < 0.01, ***P < 0.001.

## Data Availability

The analyzed data sets generated during the present study are available from the corresponding author on reasonable request.

## Abbreviations

DN: Diabetic nephropathy
ROCK1: Rho-associated coiled-coil-containing kinase 1
RTECs: Renal tubular epithelial cells
CircRNA: Circular RNA
miRNAs: microRNAs
ceRNA: competitive endogenous RNA
HG: High glucose
