# Supplementary figures and images for: Circ_0000064 promotes high glucose-induced renal tubular epithelial cells injury to facilitate diabetic nephropathy progression through miR-532-3p/ROCK1 axis
Source: BMC Endocr Disord. 2022 Mar 15;22:67. doi: 10.1186/s12902-022-00968-x (PMC8922934; doi:10.1186/s12902-022-00968-x)

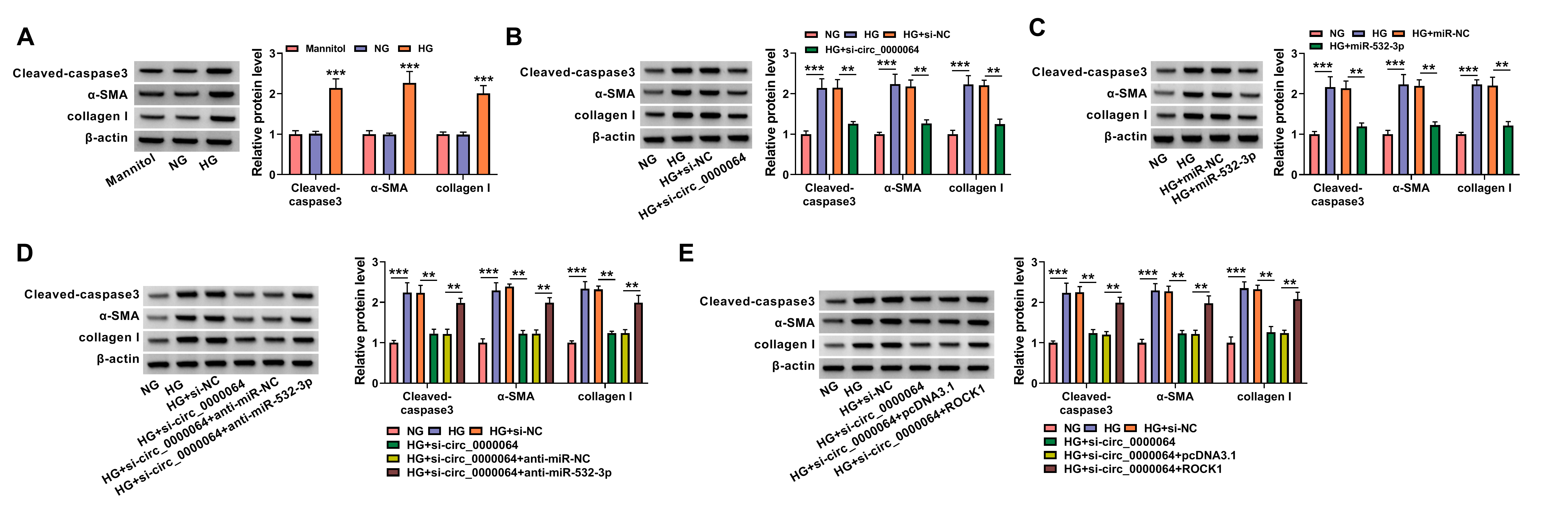

Supplement: Supplementary file 1 — Additional file 1: Supplementary Figure 1. The protein expression of cleaved-caspase3, α-SMA and collagen I. Protein expression was detected under the treatment conditions as shown in Fig. 1 (A), Fig. 3 (B), Fig. 5 (C), Fig. 6 (D) and Fig. 8 (E). **P < 0.01, ***P < 0.001. [file 12902_2022_968_MOESM1_ESM.tif]

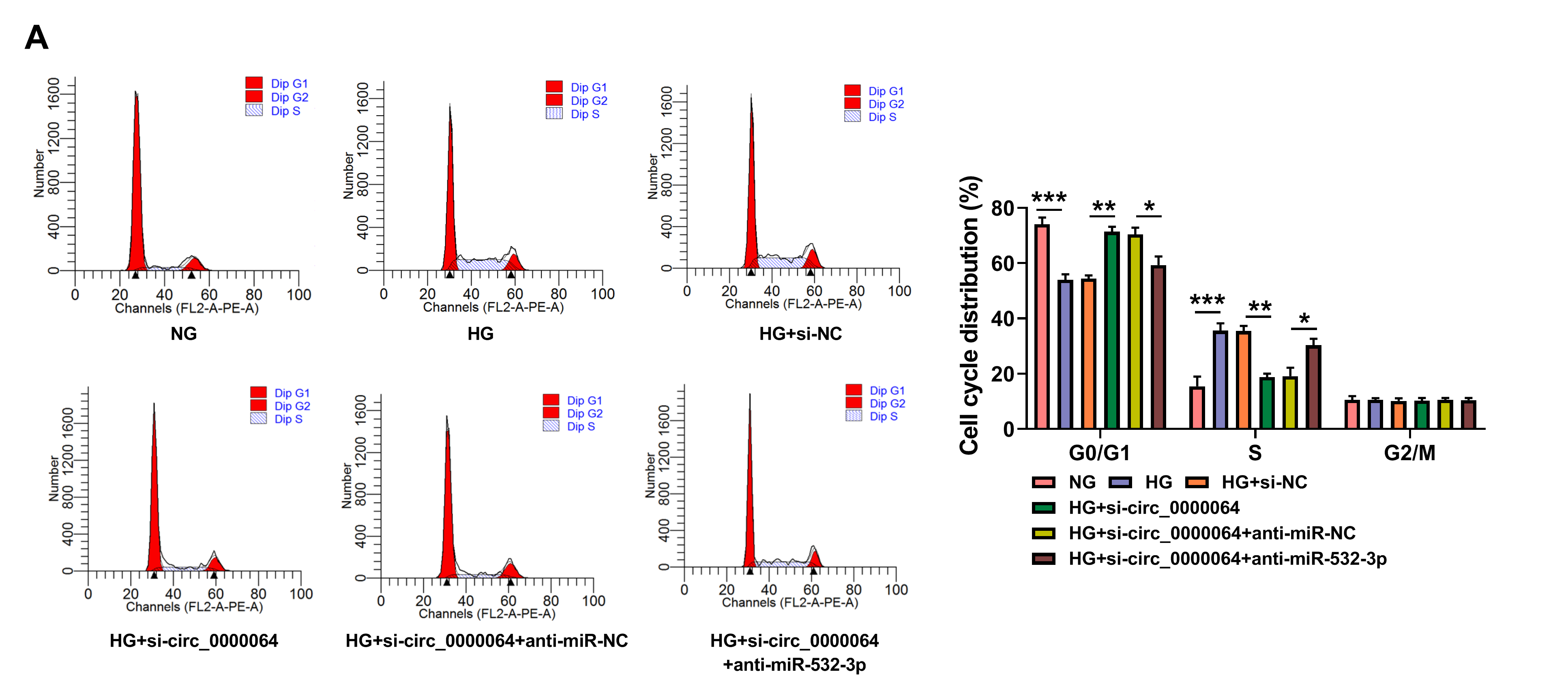

Supplement: Supplementary file 2 — Additional file 2: Supplementary Figure 2. Effects of si-circ_0000064 and anti-miR-532-3p on cell cycle. HK-2 cells were transfected with si-NC or si-circ_0000064 and then treated with HG. Cell cycle distribution was assessed by flow cytometry. *P < 0.05, **P < 0.01, ***P < 0.001. [file 12902_2022_968_MOESM2_ESM.tif]

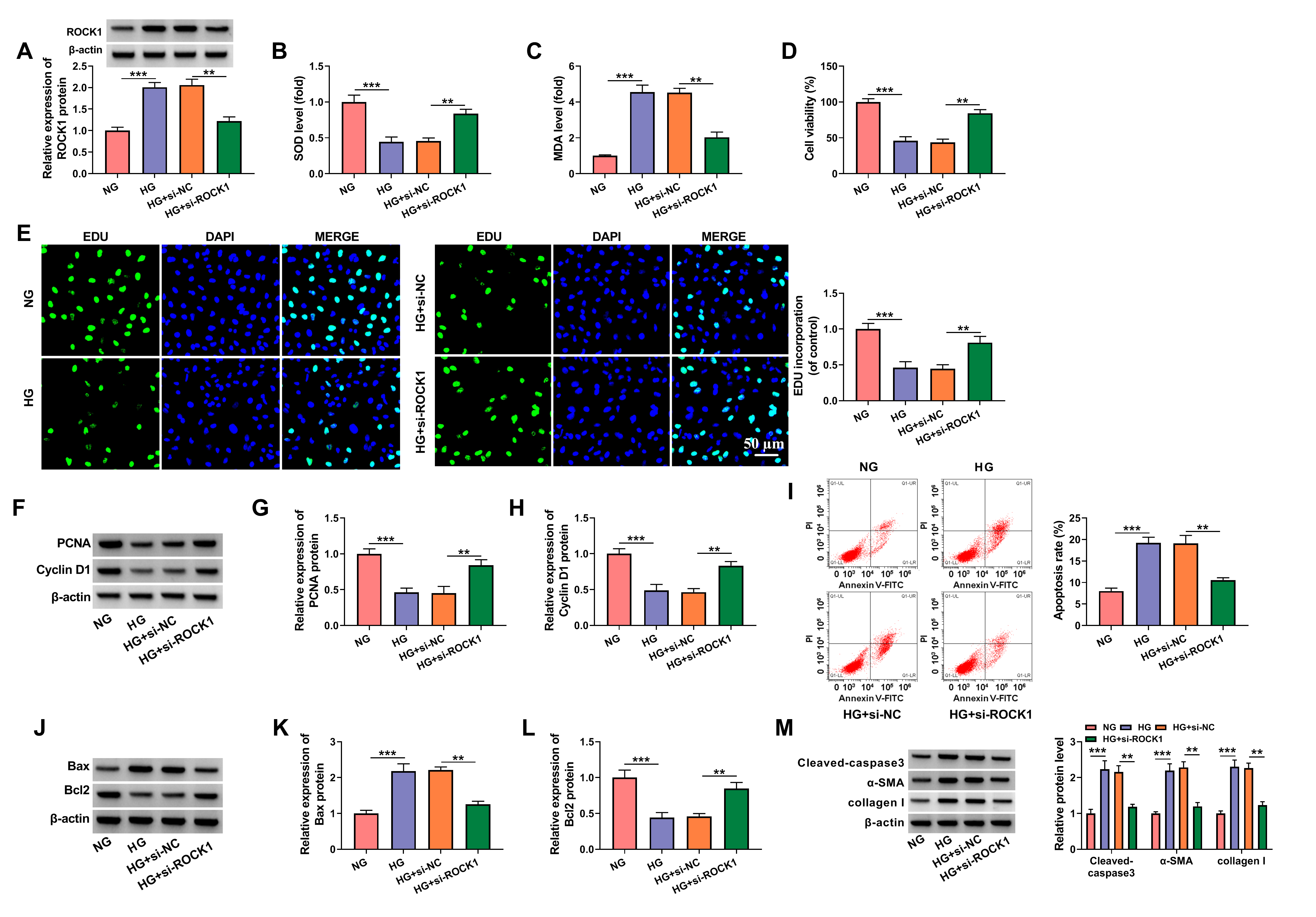

Supplement: Supplementary file 3 — Additional file 3: Supplementary Figure 3. Knockdown of ROCK1 relieved HG-induced HK-2 cells injury. HK-2 cells were transfected with si-NC or si-ROCK1 and then treated with HG. (A) The ROCK1 protein expression was detected by WB analysis. (B-C) Cell oxidative stress was analyzed by detecting SOD and MDA levels. CCK8 assay (D) and EDU assay (E) were used to assess cell proliferation. (F-H) The protein expression of PCNA and cyclin D1 was tested by WB analysis. (I) Flow cytometry was used to examine cell apoptosis rate. (J-M) The protein expression was determined by WB analysis. **P < 0.01, ***P < 0.001. [file 12902_2022_968_MOESM3_ESM.tif]
